# Prophylactic Circumferential Retinal Cryopexy to Prevent Pseudophakic Retinal Detachment after Posterior Capsule Rupture during Phacoemulsification

**DOI:** 10.1155/2015/807389

**Published:** 2015-11-30

**Authors:** T. Bertelmann, C. Heun, C. Paul, E. Bari-Kacik, W. Sekundo, S. Schulze

**Affiliations:** Department of Ophthalmology, Philipps-University Marburg, Baldingerstraße, 35043 Marburg, Germany

## Abstract

*Purpose*. To evaluate whether prophylactic circumferential retinal cryopexy (CRC) can prevent pseudophakic retinal detachment (PRD) development after posterior capsule rupture (PCR) during phacoemulsification.* Methods*. Retrospective patient chart analysis of eyes experiencing a PCR during phacoemulsification. Comparison of PRD development between eyes receiving CRC (cryo+ group) or not (cryo− group).* Results*. Overall 106 patients were analyzed, thereof 61 (58%) in the cryo+ and 45 (42%) in the cryo− group. In both clusters a total of 10 PRDs (9.4%) occurred, thereof 3 (30%) in the cryo+ as well as 7 (70%) in the cryo− group (*p* = 0.087), 79.8 ± 81.58 weeks after PCR. Relative/absolute risk reduction in CRC-treated eyes was calculated to be 68%/11%. Prophylactic CRC reduced PRD development 0.3-fold. Number needed to treat was estimated to be 9.4.* Conclusion*. Prophylactic CRC might be a useful treatment option in eyes with PCR to hamper PRD development in the further course. Further research is indicated to evaluate this beneficial effect between eyes with and without a rupture of the anterior vitreous cortex and accompanying vitreous loss in an expanding number of eyes.

## 1. Introduction 

Cataract is the most common cause of reversible vision loss in the world. Many advances have been made within the last decades to improve the surgical lens removal procedure, such as phacoemulsification technique, small incision surgery, the use of viscoelastics, and the development of intraocular lenses [1]. This ensures a less traumatic approach to the eye, reduces complication rates, and backs up rapid visual recovery in most cases [1, 2]. Therefor cataract surgery has ascended to be the most frequently performed surgical intervention in developed countries nowadays [3]. Despite these major improvements various complications, including endophthalmitis, acute corneal decompensation, raised intraocular pressure (IOP), or postsurgical cystoid macular edema, may occur [4]. Posterior capsule rupture (PCR) during the surgical maneuver is another major complication raising the risk for pseudophakic retinal detachment (PRD) in the further postsurgical course [5]. Former research described a protective effect of prophylactic circumferential retinal cryopexy (CRC) in aphakic eyes [6] or in eyes with peripheral retinal breaks [7] in respect to retinal detachment (RD) development. Thus, the purpose of this investigation was to evaluate whether prophylactic CRC after PCR during a complicated phacoemulsification procedure can prevent PRD development.

## 2. Material and Methods

A retrospective patient chart analysis was performed including all phacoemulsification cases performed at Department of Ophthalmology, Philipps-University Marburg, Germany, in which a PCR occurred during the operation between July 1996 and December 2012. To be included into this investigation, patients needed to be 40 years of age or older scheduled for a routine age-related cataract removal procedure using phacoemulsification technique. The postsurgical observation period needed to extend 2 years at a minimum. Exclusion criteria were eyes with an axial length (AL) of more than 25 mm, congenital or traumatic cataract formation, previously vitrectomized eyes, and any combination of the phacoemulsification procedure with other ocular surgical procedures, such as keratoplasty, glaucoma operations, or posterior segment surgery.

### 2.1. Statistical Analysis

Tables were prepared using Microsoft Word 2007 (Microsoft©). Statistical analysis was performed with Office Excel 2007 (Microsoft©) and SPSS Statistics 20 (IBM©). To test baseline value differences between groups, binomial distribution test and Mann-Whitney *U* test were performed. To test the effect of retinal cryocoagulation in respect to PRD rates, logistic regression was executed including cryo+/−, axial length (AL), time till cryocoagulation, and patients' age and gender as covariates. Significant results were assumed if *p* values were less than 5% (*p* < 0.05).

## 3. Results 

Overall 106 patients were included into this analysis, thereof 55 male (51.9%) and 51 female (48.1%) subjects with an overall age of 75.1 ± 8.4 years (mean value ± standard deviation). Patients were split into a cryocoagulation (cryo+) and a noncryocoagulation (cryo−) group depending on whether prophylactic CRC was performed after PCR or not. Patients' baseline characteristics of each group are displayed in Table 1.

In the cryo+ group prophylactic CRC was performed 11.6 ± 27.2 weeks after PCR. A total of 10 (9.4%) PRD occurred in both groups, thereof 3 (30%) in the cryo+ as well as 7 (70%) in the cryo− group (*p* = 0.087). Relative/absolute risk reduction in CRC-treated eyes was calculated to be 68%/11%. Prophylactic CRC reduced PRD development 0.3-fold. Number needed to treat (NNT) was estimated to be 9.4. Axial length (*p* = 0.484), time till cryocoagulation (*p* = 0.657), and patients' age (*p* = 0.394) and gender (*p* = 0.498) did not have a significant impact on PRD development.

Overall PRD occurred 79.8 ± 81.58 weeks after the eventful phacoemulsification. In all cases pars plana vitrectomy (ppV) was performed for successful RD repair.

## 4. Discussion

Major improvements in extracapsular cataract extraction (ECCE) procedures have been made within the last decades, especially the replacement of manual nuclear extraction by phacoemulsification [8]. Additionally, a stepwise improvement of the latter resulted in a further significant decrease of complication rates. Hereby, the number of posterior capsule ruptures (PCR) and anterior vitrectomies (AV) halved despite substantially increasing procedure counts [8]. PCRs were reported to occur in between 0.45% and 16% of all phacoemulsification procedures mostly dependent on surgical experience [4, 9–11] and other various risk factors [12]. Thus, PCRs remain one of the most common complications in cataract surgery with a major risk of compromised final visual outcome [4, 9]. PCRs oftentimes occur during the phacoemulsification (roughly 60%) or irrigation/aspiration (about 25%) process [4]. Accompanying vitreous loss (VL) is associated with an even poorer visual acuity (VA) outcome and typically occurs, in about 1.0% to 75% of PCR cases, during nuclear disassembly and removal [4, 11, 13].

Former reports indicated a fivefold increase of retinal detachments in pseudophakic eyes in which a PCR and VL occurred during the phacoemulsification procedure in comparison to uncomplicated cataract surgeries [4, 5]. Contrariwise, prophylactic circumferential retinal cryopexy (CRC) was successfully used in eyes prior to cataract surgery in patients prone to retinal detachments [14] and in several patients undergoing pars plana vitrectomy [15, 16]. Prophylactic CRC is also administered for various peripheral lesions like retinal breaks, tears, and others such as lattice degeneration to prevent RD development [17], if not addressable with laser photocoagulation. Thus, the question arises, whether prophylactic CRC after an eventful phacoemulsification procedure can reduce PRD development in the further course. As demonstrated herein there was a meaningful reduction in PRD development in the cryo+ group (relative/absolute risk reduction in prophylactic CRC-treated eyes of 68%/11%) although statistical significance failed. This in turn is essentially attributed to the marginal number of overall PRD developments of 9.4% (cryo+: 3 PRDs in 61 PCR cases; cryo−: 7 PRDs in 45 PCR events) in this series. According to this data, calculation of number of cases to show statistically significant differences revealed group sizes of 139/179 PCR cases in each group to reach a statistical power of 80%/90%. In particular the NNT of 9.4 cases emphasizes the benefits of prophylactic CRC in routine patient care when comparing with NNT of 25 for prophylactic warfarin intake to prevent stroke in atrial fibrillation [18] for instance. This in turn awards a positive risk-benefit profile of prophylactic CRC in PCR cases. Nevertheless there are potential risks such as macular pucker formation, proliferative vitreoretinopathy (PVR), or surgically induced scleritis [17, 19], and therefore, individual risks and benefits for each patient have to be weighted. In this regard the technique of CRC is also important and a mild CRC (just visible whitening of the retina) should be preferred over distinctive freezing [20, 21]. Alternatively 360° laser retinopexy might be another option to prevent PRD development as laser treatment is routinely used to seal peripheral retinal breaks or degenerative areas prone to RD accruement, at least if they are symptomatic [22]. So far there is no report in the literature about the efficacy of prophylactic 360° laser retinopexy in eyes with PCR during phacoemulsification. Specific complications in the anterior [23] as well as posterior segment [24] can occur and have been reported as well. Contrariwise laser retinopexy is less traumatic to the eye and therefore a prospective study using laser instead of cold for prophylactic retinal treatment is indicated.

In theory, CRC can be used to induce permanent chorioretinal scar development and thus “glue” the retina to the underlying choroid. This could be of importance after a PCR due to the anterior movement of the vitreous towards the anterior segment of the eye. This anterior shift is additionally increased after anterior vitreous cortex (AVC) rupture with vitreous loss and a consequently performed anterior vitrectomy [25]. As the vitreous cortex is attached to the peripheral retina, the anteriorly directed drive of the vitreous body causes vitreoretinal traction and can induce retinal break or tear formation and thus induce PRD development [25, 26]. Thus a prophylactic CRC seems reasonable and the data herein support its routine use.

The strength of this evaluation is, to the best of our knowledge, to be the first investigation to evaluate whether prophylactic CRC after PCR during a complicated phacoemulsification procedure can prevent PRD development. A reasonable number of patients were included and observed over a long postoperative time. The limitation is the retrospective study design. Furthermore, the effect of prophylactic CRC may differ between eyes with and without AVC rupture and accompanying vitreous loss. Performing anterior vitrectomy in these scenarios can additionally prevent PRD significantly [27]. The position of the lens implanted (sulcus ciliaris, in the bag, optic capture) and whether the eye stays (temporarily) aphakic or not [11] might also be of key interest in this regard. Due to the small number of PCR and accompanying PRD cases eligible for this evaluation within a 16.5 years' observation period, a separated and additional evaluation of these unanswered questions was not possible and would need some decades to gain enough patients. Nevertheless, these essential questions need further evaluation on a larger number of eyes affected in the future.

## Figures and Tables

**Table 1:** Baseline characteristics of patients receiving prophylactic circumferential retinal cryopexy (cryo+) or not (cryo−) after posterior capsule rupture during phacoemulsification.

	Cryo+ group (*n* = 61/58%)	Cryo− group (*n* = 45/42%)	*p* value
Gender (male/female)	31 (51%)/30 (49%)	37 (55%)/30 (45%)	0.771
Age (phacoemulsification)	75.1 ± 8.3 years	75 ± 8.6 years	0.931
Eye affected (right/left)	32 (52%)/29 (48%)	27 (60%)/18 (40%)	0.285
Axial length (mm)	23.16 ± 0.78	23.14 ± 0.96	0.977
